# Supplementary material for: The Obesogenic Gut Microbiota as a Crucial Factor Defining the Depletion of Predicted Enzyme Abundance for Vitamin B12 Synthesis in the Mouse Intestine
Source: Biomedicines. 2024 Jun 9;12(6):1280. doi: 10.3390/biomedicines12061280 (PMC11201498; doi:10.3390/biomedicines12061280)
Supplement: Supplementary file 1 [file biomedicines-12-01280-s001.zip › biomedicines-2964924-supplementary.pdf]

Supplementary Table S1. Primers used to amplify the V3-V4 region of bacterial 16S rRNA gene.

Forward Primer:	**TCGTCGGCAGCGTCAGATGTGTATAAGAGACAG**CCTACGGGAGGCAGCAG
Reverse Primer:	**GTCTCGTGGGCTCGGAGATGTGTATAAGAGACAG**GACTACAAGGGTATCTAATCC
